# Neurosurgical Operative Atlas: Spine and Peripheral Nerves

**DOI:** 10.1055/s-0038-1669395

**Published:** 2018-08-13

**Authors:** J. Bahm

**Affiliations:** 1 Euregio Reconstructive Microsurgery Unit, Franziskus Hospital, Aachen, Germany

**Neurosurgical Operative Atlas: Spine and Peripheral Nerves edited by C.E. Wolfla and D.K. Resnick [Thieme 2017, 3rd edition]**

The 2017 third edition of this operative atlas brings together a lot of skilled spine surgeons with peripheral nerve surgeons who deal with regular PNS procedures.

It is a well-conceived and well-structured book where every chapter highlights mandatory knowledge, surgical steps, material requirements, altogether with beautiful illustrations and photos of surgical details. As announced in the preface, it addresses both experienced colleagues (looking for a “pearl” detail) and residents who want to review “the night before” a procedure they will accompany the next working day.

As spine surgery is rapidly evolving, due to the development of minimal invasive approaches, instrumentation refinements, and overall growing technical support (endoscopy, robotics)], this type of atlas will again have to change—but here, it offers the interested reader a concise and clear insight on how regular spine surgery is performed today.

Part IV is dedicated to the peripheral nerves, and not only reviews common nerve decompression, surgical exposures of peripheral nerves of the upper and lower extremity, but also insights into the specific reconstruction of brachial plexus lesions and meralgia paresthetica, concluding with an overview of the principles for peripheral nerve repair in general and for graft harvesting.

This book contains over 500 pages, and the reader gathers a lot of information from the text, specific illustrations, and clear figures. It is a beautiful atlas, not a textbook. You will dive in to seek for a specific exposure or technique, and then continue to look around. You will certainly understand the actual “state of the art,” and understand where the spine surgery stands and what direction it moves.

**Atlas of Peripheral Regional Anesthesia: Anatomy and Techniques edited by G. Meier and J. Buettner [Thieme 2016, 3rd edition]**

Regional anesthesia completely changed after the progressive arrival of ultrasound imaging techniques. Therefore, this atlas starts with the basics of ultrasound guidance: the equipment, the needle approach, the continuous block technique. It then runs into the upper and lower limb, specifically deals with the child patient and concludes with more general insights including complications, analgosedation, and continuous blocks. In the limb section, one moves in topographic order from proximal to distal, and every location is presented first by topographic anatomy, and then the approach, its indications, sensory and motor effects, and possible complications. Thereby, it serves daily routine chapter by chapter and presents all aspects for the practitioner, either beginner or experienced.

The book had its success through previous German and later translated editions, and kept its value by the confrontation of anatomic figures with the ultrasound appearance, before describing how the needle should be inserted and how the local anesthetics should be dosed.

Interestingly, not all these techniques became standard procedures; some approaches are country-specific (e.g., the continuous peripheral nerve blocks we use in Germany for patients suffering from chronic neuropathic pain which we treat in a 1 to 3 weeks hospital stay with interdisciplinary strategy including the anesthetist and surgeon, the physio- and occupational therapist, and the psychologist).

The presented techniques will still evolve with time, indications or dosage might change—but this third edition is a landmark atlas for all those involved in peripheral regional anesthesia techniques, and of course anesthetists, upper limb surgeons, nurses, and technicians all involved in these precise procedures, which highly alleviated limb surgery and related pain management.

